# Dr. I. F. Meigs’ Case of Inflammation and Necrosis of the Femur, with Femoral Abscess, Terminating Fatally on the Thirtieth Day

**Published:** 1842-08-27

**Authors:** J. F. Meigs


### MEDICAL EXAMINER.

NEW SERIES.

No. 35.] PHILADELPHIA, AUGUST 27, 1842. [Vol. I.

### *Case of Inflammation and Necrosis of the Femur, with Femoral Abscess, terminating fatally on the thirtieth day.*

By J. F. Meigs, M. D.

A. B., an American, aged about twenty-six years, of large frame, but sallow, unhealthy appearance, was attacked on the 6th July, 1842, with severe pain in the inferior part of the right thigh, extending at times to the knee-joint. Of the cause of the attack the patient was quite ignorant; he had met with neither fall nor blow; nor was he labouring under syphilis or rheumatism. Some of his friends were inclined to ascribe it to the position required by his occupation, that of cutter-out in a shoe manufactory. While at work he was obliged to stand before a counter, and as he was thus employed during the whole day, it is possible that this may have been the immediate cause, though the remote cause undoubtedly lay in his feeble constitution. What may perhaps render this more probable is, that one of his fellow-workmen died of the same disease a few months previous.

The pain, which was at first intermittent, soon became constant; from being dull and aching it became very severe, and at times excruciating; he lost the use of the limb; the leg was flexed upon the thigh, and could be extended only with great difficulty; he lost his appetite and became emaciated; fever set in; and in the last week of July, when my father was called to see him, his condition was as follows:

The pain was referred to the neighbourhood of the bone; slight pressure produced no inconvenience, while, if firm, it could not be borne; there was no swelling or soreness about the knee-joint; there was no tumefaction of the thigh; and, in fact, but for the pain, and a slight hardness of the tissues, no disease could have been suspected. Though lying down and affirming that he could not walk, he was enabled, after gently rubbing the part for a time, to get up and walk around the bed. A blister was applied to the inside of the thigh just above the knee; the diet to be nutritious but simple; and he was to take an anodyne at night.

On the 1st of August, when I saw him, he was labouring under all the symptoms of violent irritative fever, or what is described as the shock to the system. The countenance was haggard and pinched; the skin pale and sallow; distribution of heat unequal; while the head was hot and dry, his hands and feet were cold, and covered with a clammy perspiration; pulse frequent, from 120 to 140, small, and very compressible; tongue dry and brown; respiration rapid or irregular; urine scanty and high coloured, and the bowels torpid. He had no appetite, and had evening exacerbations of fever.

Upon a careful examination of the thigh, the lower internal part was found swollen and hard, and the posterior surface also was firmer than natural. The outline of the vastus internus muscle could be distinctly traced from the tumefaction of its tissue. The skin and subcutaneous tissue felt harder than usual around the whole thigh, and the former presented the peculiar marbled appearance seen in inflammation of the lymphatics. Slight pressure caused no pain, but when firmer, the soreness was very great. There was no appearance of disease about the knee-joint. His decubitus was peculiar, and one which I believe is always assumed by a patient with abscess in the lower part of the thigh. He lay upon his back, with the thigh slightly flexed upon the pelvis, the leg upon the thigh, and the whole limb resting upon its outer surface, by which position the muscles inflamed were relaxed, and the weight of the member borne upon the strong fascia of the vastus internus muscle. From this he never willingly moved. A large poultice of flaxseed meal was applied to the thigh, and directions given to renew it frequently ; five grains of ammon. carbonat., with wine whey every two hours, was prescribed, and the diet to consist of chicken soup and farinaceous substances. His bowels were kept open by enemata.

In the night of the 2d of August he was attacked with delirium, very much resembling mania-a-potu, of the form called by Dupuytren Traumatic Delirium.

From this period he grew rapidly worse. The delirium became constant; the head was very hot; the extremities cool; the countenance pinched and cadaverous; the pulse grew weaker and more rapid; he had frequent involuntary stools; the pupils were contracted; he lost the power of external sensation in a great measure; and notwithstanding the use of large doses of carbonate of ammonia, with beef tea and brandy punch, he sank and died early on the morning of the 5th of August.

*Post-Mortem.*

Upon a comparison of the two limbs it was evident that the right thigh was tumefied in its lower third, principally upon the interior semi-circumference; and the region of the vastus internus muscle was swollen so as to project above the surrounding parts. No fluctuation whatever could be detected after the most careful search.

An incision was made through the vastus internus muscle, commencing about four inches above the knee, and extending downwards to the tibia. When within a short distance of the bone, at the upper extremity of the cut, an abscess was opened, containing some three or four ounces of yellow, nearly inodorous pus. The walls of the abscess were tense and unyielding, and this explained readily the absence of fluctuation. The finger introduced into the opening made into the abscess, passed downwards for a distance of about two and a half inches, and around two-thirds of the shaft of the bone, so that nearly three inches of the femur were stripped of its muscular attachments and completely bathed in pus. After removing the soft parts from the bone, and examining it carefully, the periosteum, from the line of attachment of the articular cartilages, to about three inches and a half above this line, was seen to be either quite detached from the bone, or easily separable with the handle of the knife. It was very much thickened, and highly vascular. Between its laminæ were placed many small fragments of bones. These were at first thought to be portions detached from the femur, but upon a second examination I concluded them to be new deposits from the periosteum, intended, no doubt, like the provisional callus of fractured bones, to form an exterior shell to support the limb, while the diseased portion within should undergo the changes necessary for its removal by absorption or elimination. On sawing longitudinally through the femur we found its cancellated structure filled with pus; and upon tapping it, it gave out the clear ringing sound so characteristic of necrosis.

On account of objections made by the friends of the patient, we could not prosecute the examination far enough to determine the precise point where the disease terminated. The knee-joint was quite healthy, except that it contained a small quantity of turbid serum.

*Observations.*

The most important object, in a case of this kind, is to determine at once the diagnosis, because of the great importance of the treatment during the early stage. In the early stage of such an attack, it is very apt to be mistaken for rheumatism or neuralgia; nor is it very easy to distinguish it from the former; however the character of the fever, the great rapidity of the pulse, the irregular distribution of heat, the expression of the countenance, which is usually very anxious, the character of the pain, and its situation, and the peculiar decubitus, will in a very few days lead one to the true nature of the illness. It is important to make this distinction, for if such a disease can possibly be arrested in the early stage it should be done; and the only probable chance is a full and free use of all the antiphlogistic measures in the very beginning of the attack. After suppuration has taken place and can be clearly proved, the question arises, shall an opening be made? To this the great weight of authority replies in the affirmative, and I doubt very much whether in a case like the above, where, though fluctuation cannot be felt, every other symptom indicates its presence, a surgeon would not be quite justifiable in making an incision to the bone, and thus, at least, relieving the extreme tension of the part, to which may be ascribed (as is the case in paronychia) the excessive constitutional irritation under which the patient labours. Another very interesting feature in this case is the great rapidity with which the disease advanced, terminating in suppuration, necrosis of the bone, and death in thirty days from the attack.

